# Asking More of Our EHR Systems to Improve Outcomes for Pediatric Patients

**DOI:** 10.3389/fphar.2020.00253

**Published:** 2020-03-12

**Authors:** Jeffrey S. Barrett

**Affiliations:** Quantitative Sciences, Bill & Melinda Gates Medical Research Institute, Cambridge, MA, United States

**Keywords:** EHRs, precision dosing, decision support, dashboards, pediatric patients, hospital

## Introduction

The widespread global interest in electronic health records (EHRs) has created an impression that the requisite engagement of caregivers, providers, patients, and the institutions that rely on EHRs is well-established and in place (Pagliari et al., 2007). Meaningful Use regulation is similarly well-intended but also gives the impression that proper linkages are in place and that the relationships are optimized to create value. The point made a decade ago that “inevitability does not mean easy transition” (Blumenthal and Tavenner, 2010) is still true today. For pediatrics this is especially true. While recent legislation has obligated pharmaceutical sponsors and manufacturers (PhRMA) to be more proactive in the planning, design, and conduct of pediatric clinical trials in populations that would stand to benefit from potential new medicines, the totality of data that would support meaningful dosing guidance as well as long-term safety experience is still lacking (Barrett et al., 2018). As the assumption that adult and pediatric disease progression is similar is often not valid, bridging experiments and trials are likewise not always appropriate with the default scenario that dosing in children is heavily reliant on the adult experience. EHRs have the potential to generate meaningful real-world data (RWD) in children if a mechanism can be adopted that both captures the relevant outcome data and decision support systems built on such data become a part of the practice of medicine and not just a reference.

## EHRs Past, Present, and Future

Since the 1960s, medical records were primarily paper-base with various raw data listings capturing test results and procedures in addition to prescribing (medicine orders) and billing information. The source of these record was often not centralized and based on chart-based collection managed by the group collecting and responsible for the data (e.g., Pharmacy/formulary, Pathology, diagnostics). In addition, what we now refer to as personal health information (PHI) including social security numbers, names, and addresses was scattered all of the source documents. Lockheed's initial EHR offering developed in the 1960's became the anchor for future systems developed in the 1980's with the support and guidance from the Institute of Medicine (IoM). Since the early 2000's there has been great motivation to convert all paper-based records to EHRs with incentive provided by the Office of the National Coordinator (ONC) of Health Information Technology (IT). The subsequent Health Information Technology for Economic and Clinical Health Act (HITECH) promoted the concept of “meaningful use” criteria with the intention of providing definitive outputs and incentives for EHR-adopting institutions (CDC, 2019).

On a personal level I was fortunate to receive grant funding from the American Reinvestment & Recovery Act (ARRA) which was enacted on February 17, 2009. As ARRA sought, in part, to provide opportunities to demonstrated meaningful use for electronic health records under the umbrella of the “Health Information Technology for Economic and Clinical Health (HITECH) Act,” our grant was focused on developing prototype drug dashboards which provided dosing guidance for therapeutic agents for which therapeutic dug monitoring was employed to manage pharmacotherapy (Barrett et al., 2008). While there was engagement with the hospital governance committees on the development of the dashboard prototypes the incentives for implementing the systems were not able to convince these committees on adopting or even testing the prototypes in actual patients despite retrospective evaluation of their performance and acknowledgment of their accuracy (Barrett et al., 2008). It became apparent that the value of the record systems as billing and documentation of services rendered represented too great a value to overcome the potential benefit of the prototype systems which required prospective clinical validation to fully demonstrate their clinical value. While the HER systems offer great potential clinical functionality, their implementation is still guided primarily by IT and financial motivations. Hence, the potential to guide the practice of medicine has been there for some time, though the habit and culture of doing so has not been in place.

## Hospital Culture for Guidance and Therapeutics Standards

Most hospitals maintain some level of hierarchy regarding therapeutic decision making particularly pediatric institutions where formulary and standards recommendations are often made in the absence of actual pediatric-specific labeling for old and new drugs. For new drugs in particular, while there may be great motivation to initiate therapy with the new agent in children, there is often a prevailing conservativism especially if the sponsor has little data to support pediatric claims or if there is only a rationale with no claims or data available. For new drug candidates this is often under the jurisdiction of the drug use evaluation (DUE) or similar committee and/or the therapeutic standards or similar committee (TSC) with the distinction being the former is more pharmacy-based and informed about clinical pharmacology and the latter is more physician-based with emphasis on clinical outcomes. Both are intimately concerned with patient safety and typically, the DUE will provide recommendations for the TSC's approval.

Simply building decision support systems or outcome-based dashboards is not enough. There is a long history of efforts to create such tools, but they have not been sustainable even when internal champions exist at the host institutions (Jelliffe, 1968; Barrett et al., 2008; Jelliffe et al., 2009; Felton et al., 2014; Mould et al., 2014, 2018; Neely, 2017). This is also not an issue of regulatory compliance or providing guidance outside the drug label though this is often cited as a barrier for adoption.

## Continued Lack of Guidance for Pediatrics

While a premium is placed on the safe use of medicines in children, the data useful to guide safe practices is seldom discussed in the proper context. The historical argument is that we need more studies in children with pediatric-specific formulations and better dosing guidance derived from pediatric trials and we need to more closely monitor off-label usage (Barrett et al., 2011). Thus, we keep looking to the pharmaceutical industry to fill in gaps in our knowledge. It is clear that pharmaceutical sponsors still struggle with demonstrating similarity of disease progression (or not) and the subject of pediatric extrapolation (Barrett et al., 2018) continues to be a topic of interest among industry and regulatory scientists. As real-world data (RWD) sources are often lacking, EHRs have been discussed as one potential source for such analyses. More importantly however, EHRs are not considered as sources of information from which dosing guidance can be derived or even modified. Why not?

Adult medicine will always have the benefit of more choices with respect to medicine per condition and suitable data on pharmacokinetics, pharmacodynamics, and pharmacogenomics based on a marketplace economy that drives the industry and the clinical development plans of pharmaceutical sponsors. Likewise, the current paradigm for pediatric drug development is tied to a decision tree that relies heavily on adult experience regarding the therapeutic window in aggregate and extrapolation approaches leveraging the adult data (Barrett et al., 2018). Pediatric doses are calculated mostly using body weight to produce a dose for the individual child though oncology still uses body surface area to a large extent. This is not true personalization of course as the dose suggested (usually in mg/kg) can be the same for a large age range of children. More recent practice with staggered dosing across age-weight bands is a step in the right direction but still stops short of individual dosing guidance.

Precision medicine in pediatrics will require an appropriate evidence base (particularly for unlicensed and off label medications), with relevant patient-specific data (e.g., genotype, environmental, and lifestyle data) added to guide both medication selection and the dose required (Hawcutt et al., 2016). The choice of the source of this data should not be the issue as long as it possesses the appropriate information value.

## Discussion

**Figure 1:**
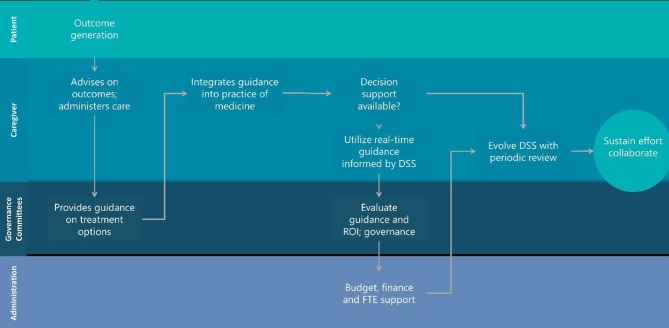
Hospital landscape for sustainable decision support engagement that evolves with the practice of medicine during habitual utilization.

What would it look like to realize the work of Mould et al. (Barrett et al., 2008; Mould et al., 2014, 2018; Hawcutt et al., 2016; Strik et al., 2018, 2019) in a more meaningful and sustainable way? Fundamentally, this will require more engaged hospital governance committees comfortable with technology, underlying models and the quality of the data collected in conjunction with a culture that is more open to seeing technology built on EHRs as an extension of their practice and not a mere record keeping or billing service. Figure 1 describes a workflow proposal more consistent with establishing clinical value and return on investment for dashboard-informed EHRs with commitment to evolve and maintain the system and capture the relevant outcomes for hospitalized children. We can't keep looking to PhRMA or regulators to solve this. It is neither their problem nor is the scope of the work to be done in a place where their experience is valuable. The practice of medicine for children has to change and confidence with a new tool set must evolve to the point where precision dosing for children is an expectation and not an exception.

## Author Contributions

The author confirms being the sole contributor of this work and has approved it for publication.

### Conflict of Interest

The author declares that the research was conducted in the absence of any commercial or financial relationships that could be construed as a potential conflict of interest.
